# Combined bio-chemical fertilizers ameliorate agro-biochemical attributes of black cumin (*Nigella sativa* L.)

**DOI:** 10.1038/s41598-021-90731-4

**Published:** 2021-05-31

**Authors:** Samira Moradzadeh, Sina Siavash Moghaddam, Amir Rahimi, Latifeh Pourakbar, R. Z. Sayyed

**Affiliations:** 1 Department of Plant Production and Genetics, Faculty of Agriculture, Urmia University, Urmia, Iran; 2 Department of Biology, Faculty of Science, Urmia University, Urmia, Iran; 3 Department of Microbiology, PSGVP Mandal’s Arts, Science, and Commerce College, Shahada, Maharashtra 425409 India

**Keywords:** Biochemistry, Plant sciences

## Abstract

*Nigella sativa* L. is a medicinal plant with extensive, nutritional, pharmaceutical, and health applications. Nowadays, reducing the application of chemical fertilizers (synthetic fertilizers) is one of the main goals of sustainable agriculture to allow the production of safe crops. Therefore, the combined effect of urea and biofertilizers was studied on the quantitative and qualitative traits of *N. sativa* L. in a randomized complete block design with 10 treatments and three replications. The treatments included control (no fertilization), U (100% chemical fertilizer as urea at 53.3 kg ha^−1^, Nb (Biofertilizer, Azotobacter vinelandii), Pb (Biofertilizer, Pantoea agglomerans and Pseudomonas putida), Kb (Biofertilizer, Bacillus spp.), NPKb (NPK, biofertilizer), Nb + 50% U, Pb + 50% U, Kb + 50%U and NPKb + 50% U. The NPK(b) + U50% was related to the highest quantity of plant height, branch diameter, capsule (follicle) number per plant, auxiliary branches, seed yield per plant, thousand-seed weight, essential oil content, total phenolic compounds, flavonoid content, DPPH free radical scavenging, nitric oxide (NO) radical scavenging, superoxide radical scavenging, chain-breaking activity, phosphorus content, and potassium content, along with U for the highest biological yield and (Pb) + U50% for the highest essential oil percentage which is close to (NPKb) + U50%. The lowest value was observed in all traits related to the control treatment except for branch diameter that was related to (NPKb). Hence, the application of (NPKb) + U50% as bio-chemical fertilizers improved *N. sativa* L. Traits, so it can be recommended.

## Introduction

The detrimental impacts of chemical drugs have drawn the attention of medical circles to herbal drugs. On the other hand, since the overuse of chemical pesticides and fertilizers may adversely influence the quantity and quality of the active ingredients in medicinal plants, many pharmaceutical companies prefer raw materials produced by agrochemical-free farming or organic farming practices^1^. Besides the economic value of medicinal plants, they are adaptive to organic farming practices^2^. So, the adverse effects on their medicinal quality can be alleviated by producing them by organic practices^3^. Extensive efforts have focused on finding appropriate solutions for improving soil quality, agricultural products, and the removal of pollutants. Indeed, new farming techniques are required to mitigate these environmental hazards and enhance crop yields. One of these techniques is the evaluation of living and active soil communities to identify beneficial soil microorganisms and use them as biofertilizers^4^. Chemical fertilizers may pose many problems if they are not applied properly, such as the reduction of plant response to fertilizers, environmental problems, adverse effects on crop and food quality, the loss of soil fertility, the contamination of water supply sources, which would endanger human health, the depletion of non-renewable resources, such as phosphate rocks, and eventually the reduction of plant resistance to pests and diseases^5–7^. A fundamental principle in sustainable agriculture is the use of biofertilizers in agricultural ecosystems to significantly reduce the application of chemical fertilizer inputs^7^.

*Nigella sativa * L. is an annual plant belonging to the Ranunculaceae family that grows to the height of 60–70 cm. Its leaves are gray-green with thread-like cuttings, its flowers are colored white to blue, and its fruits are in the form of follicles containing numerous black and aromatic seeds. The seeds contain 40% fixed oil and about 1.4% essential oil and are medicinally used as carminative, cathartic, milk-promoter, anti-constipation, and sexual promoter in men. Although it is a wild species, it is cultivated in some parts of Europe, Eastern Asia, and some regions of Iran^8,9^. The biofertilizer Azotobarvar-1 (containing nitrogen-fixing bacteria from the genus *Azotobacter*) is a voluntary molecular nitrogen fixer that is capable of synthesizing biologically active compounds in the root zone including nicotinic acid, pantothenic acid, biotin, vitamin B, auxins, and gibberellins^10^. These compounds are involved in root system development and influence crop yields and soil characteristics by improving water and nutrient uptake and nitrogen (N) biofixation. *Azotobacter* can also produce plant anti-pathogenic compounds and is involved in disease control^11^. Phosphate biofertilizer Barvar-2 contains two phosphate solubilizing bacteria from *Bacillus* and *Pseudomonas* species that secrete organic acids and acid phosphatase, thereby converting insoluble P content of soil (especially in calcium-rich regions) into the soluble forms that are absorbable by plants^12^. Biofertilizers, indeed, contain various free-living microorganisms^94^, that can convert macronutrients from unavailable into available forms by biological processes and improve root system development and seed germination^13^.

Due to the increasing population of the world and also the limitation of the area under cultivation, it seems necessary to increase agricultural production by considering soil fertility and environmental hazards^14^. Biofertilizers include nitrogen fixers, phosphorus potassium, and sulfur solubilizers, mycorrhiza, Trichoderma, siderophores, etc. Biofertilizers can be an appropriate alternative to chemical fertilizers^7^. Biofertilizers, e.g., *Azotobacter*, can improve seed vigor and stimulate the plant defense system as secondary metabolites^15^.

Due to the deleterious impact of chemical fertilizers and to reduce their application for the sake of the sustainability of plant production and environmental protection, this study aimed to investigate the combined effects of bio-chemical fertilizers on biological yield, essential oil percentage and yield, antioxidant activity, and nutrient contents of black cumin.

## Results

The results of the analysis of variance (ANOVA) revealed the significance of morphological parameters and essential oil in leaves at the *p* < 0.01 level (Table 1).

**Table 1:** The results of analysis of variance for the effect treatment on morphological traits.

Sources of variations	df	Plant Height (cm)	Branches diameter (cm)	Capsule No./plant (no)	Auxiliary branches (no)	Seed yield/plant (g)	1000-seed weight (gr)	Biological yield/plant (g m^−2^)	Harvest index (%)	Essential oil percentage (%)	Essential oil yield (kg ha^−1^)
Block	2	1.381^ ns^	0.005^ ns^	0.833^ ns^	0.022^ ns^	0.006^ ns^	0.003^ ns^	0.070^ ns^	0.418^ ns^	0.0002^ ns^	0.044^ ns^
Treatment	9	8.790**	0.796**	21.392**	2.457**	1.863**	0.025**	10.273**	42.321**	0.036**	4.474**
Error	18	0.568	0.139	0.425	0.050	0.010	0.002	0.084	1.102	0.0002	0.021
Coefficient of variations		2.599	2.539	3.307	7.095	2.707	1.700	2.439	3.357	3.208	4.108

*Ns* non-significance; *significance at the *p* ≤ 0.05 level; **significance at the *p* ≤ 0.01 level.

### Plant height and branches diameter

**Figure 1:**
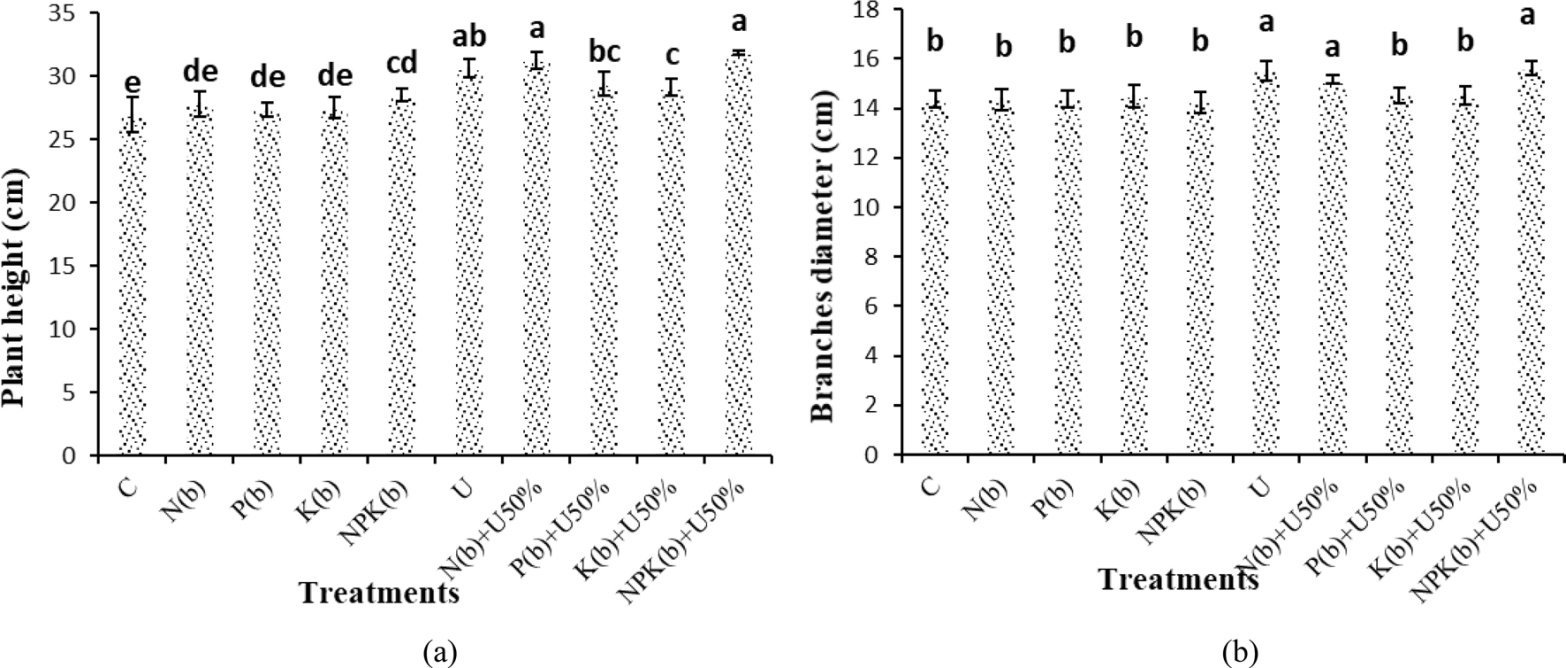
Means comparison for plant height and branch diameter of *Nigella sativa* L. as influenced by biofertilizers and urea. Dissimilar letters show significant differences at the *p* ≤ 0.01 level. Control (C), U Chemical urea, Nb (biological N), Pb (biological P), Kb (biological K, NPKb (mixed biological NPK), Nb + U50%, Pb + U50%, Kb + U50%, and NPKb + U50%.

The highest plant height (31.80 cm) was obtained from the plants inoculated with (NPKb) + U50% vis-à-vis the control plants (26.93 cm). The treatment of (NPKb) + U50% significantly differed from all the treatments except (Nb) + U50% and U. The comparison of the means for the treatments showed that (NPKb) + U50% and (Nb) + U50% increased plant height by 18.06% and 15.84% versus the control (Fig. 1a).

The highest and lowest branch diameters (15.62 and 14.23 cm) were obtained from the plants treated with (NPKb) + U50% and those treated with (NPKb), respectively. The plants inoculated with (NPKb) + U50% and U exhibited 9.71% and 9.01% higher branch diameter than the plants treated with (NPKb), respectively. There was no significant difference between (NPKb) + U50%, (Nb) + U50% and U treatments (Fig. 1b).

### Capsule (follicle) number per plant and number of auxiliary branches

**Figure 2:**
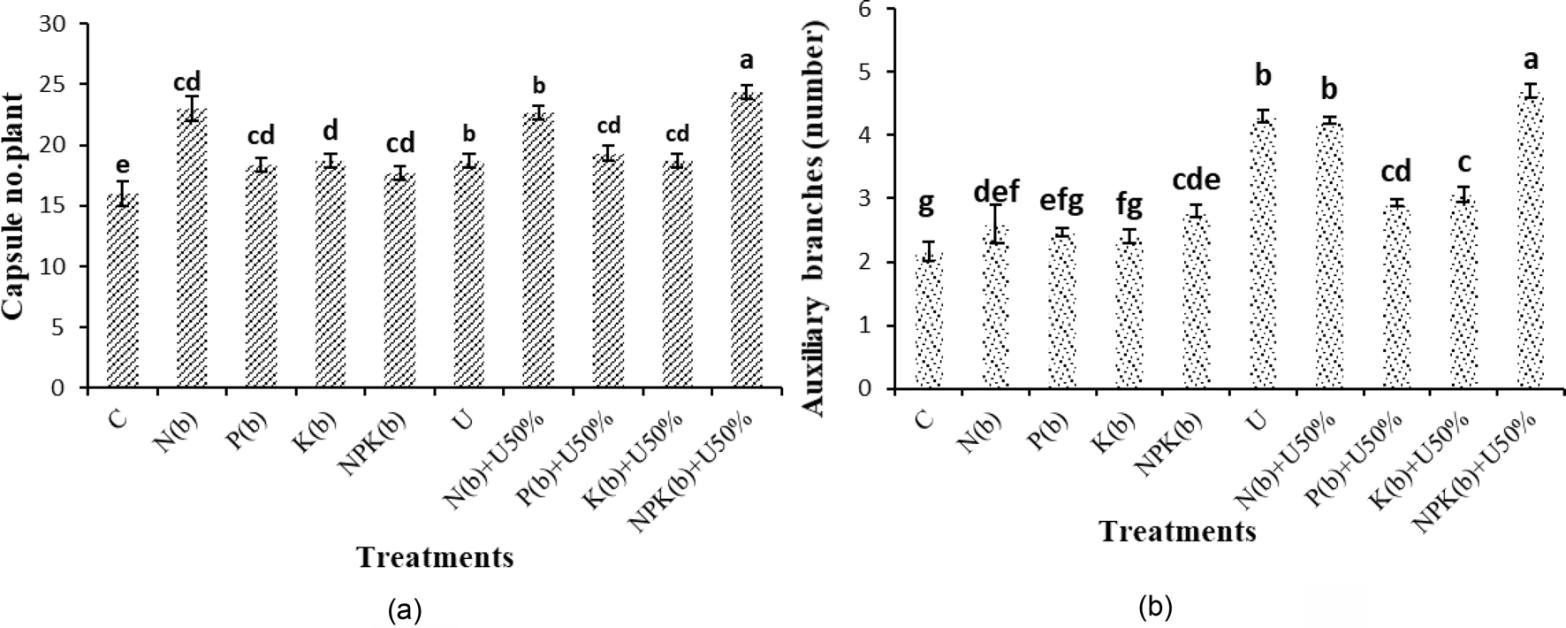
Means comparison for capsule number per plant and number of auxiliary branches of *Nigella sativa* L. as influenced by biofertilizers and urea. Dissimilar letters show significant differences at the *p* ≤ 0.01 level. Control (C), U Chemical urea, Nb (biological N), Pb (biological P), Kb (biological K, NPKb (mixed biological NPK), Nb + U50%, Pb + U50%, Kb + U50%, and NPKb + U50%.

The plants treated with (NPKb) + U50% produced the highest number of capsules per plant (24.33 capsules) as compared to 16 capsules obtained from the control plants. The plants treated with (NPKb) + U50% and U produced 52.08% and 43.75% more capsules per plant than the control, respectively (Fig. 2a).

The highest number of auxiliary branches (4.70 branches) was observed in the plants treated with (NPKb) + U50% and the lowest (2.17 branches) in the control. The treatments of (NPKb) + U50% and U increased the number of auxiliary branches by 116.92% and 98.46% versus the control, respectively (Fig. 2b).

### Seed yield per plant and thousand-seed weight

**Figure 3:**
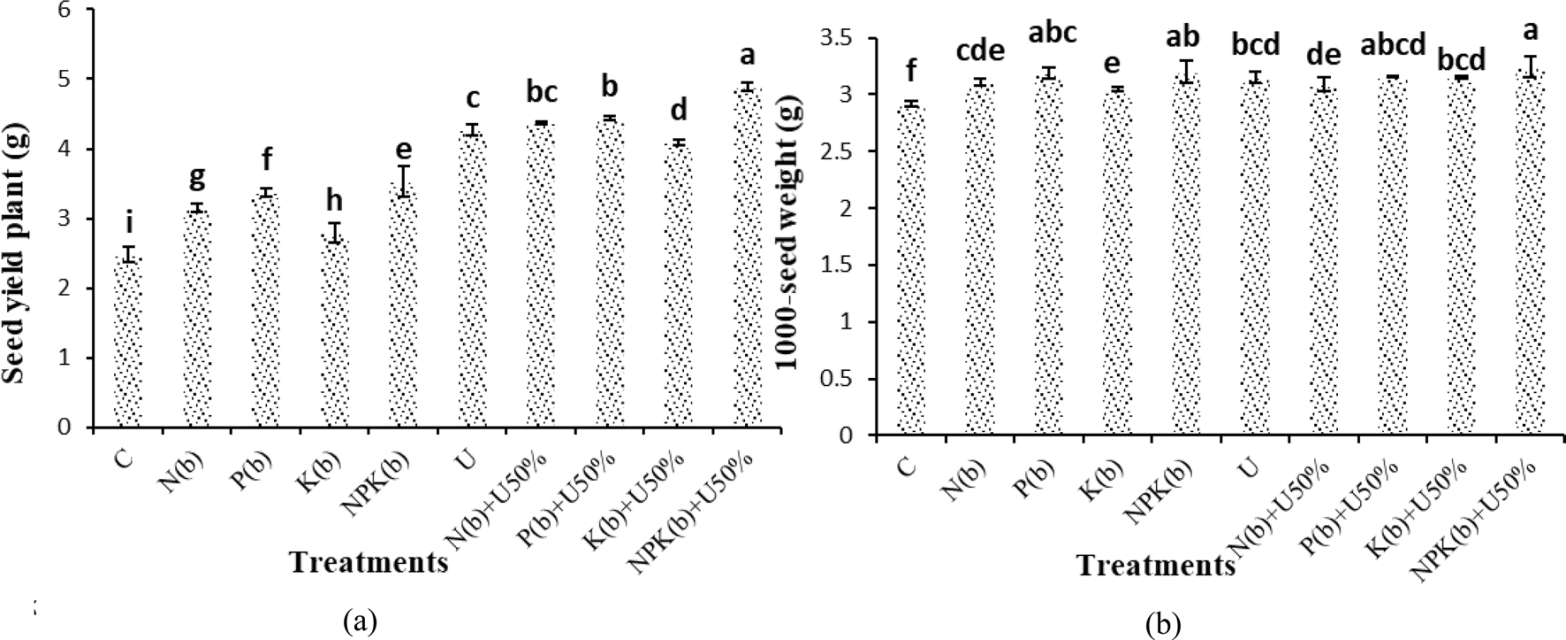
Means comparison for seed yield per plant and 1000-seed weight of *Nigella sativa* L. as influenced by biofertilizers and urea. Dissimilar letters show significant differences at the *p* ≤ 0.01 level. Control (C), U Chemical urea, Nb (biological N), Pb (biological P), Kb (biological K, NPKb (mixed biological NPK), Nb + U50%, Pb + U50%, Kb + U50%, and NPKb + U50%.

The highest and lowest seed yields per plant were observed in the treatment of (NPKb) + U50% (4.88 g m^−2^) and the control (2.48 g m^−2^), respectively. The treatment of (NPKb) + U50% had a statistically significant difference from all other treatments. The plants treated with (NPKb) + U50% and those treated with (Pb) + U50% showed 96.67% and 79.20% higher seed yield per plant than the control (Fig. 3a).

The treatment of (NPKb) + U50% was related to the highest 1000-seed weight of 3.24 g as compared to the control plants (2.92 g). The treatment of (NPKb) + U50% did not differ from the treatments of (NPKb), U, (Pb) and (Pb) + U50% significantly. The increase in the 1000-seed weight of the plants treated with (NPKb) + U50% and (NPKb) was 10.95% and 9.70% vis-à-vis the control, respectively (Fig. 3b).

### Estimation of harvest index and essential oil percentage

**Figure 4:**
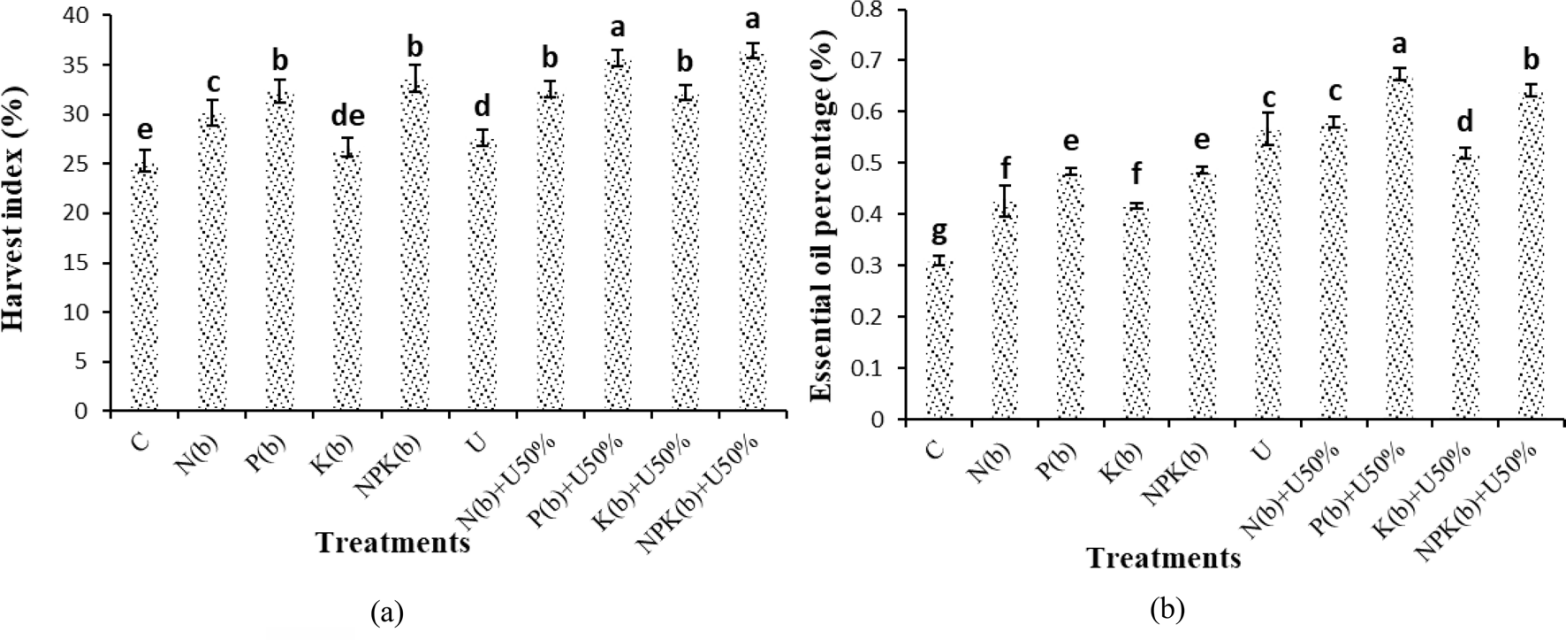
Means comparison for Essential oil percentage and Harvest index per ha of *Nigella sativa* L. as influenced by biofertilizers and urea. Dissimilar letters show significant differences at the *p* < 0.01 level. Control (C), U Chemical urea, Nb (biological N), Pb (biological P), Kb (biological K, NPKb (mixed biological NPK), Nb + U50%, Pb + U50%, Kb + U50%, and NPKb + U50%.

The treatment of (NPKb) + U50% and the control showed the highest and lowest harvest index of 36.45 and 25.31%, respectively. The treatment of (NPKb) + U50% did not show a significant difference from the treatment of (Pb) + U50%. Furthermore, (NPKb) + U50% and (Pb) + U50% increased harvest index versus the control by 44.02% and 41.01%, respectively (Fig. 4a).

The highest essential oil percentage was observed in (Pb) + U50% (0.67%) and the lowest essential oil was obtained from the control (0.31%). The (Pb) + U50% treatment was significantly different from the other treatments. The (Pb) + U50% treatment and the (NPKb) + U50% treatment increased the essential oil percentage by 117.02 and 106.98%, respectively (Fig. 4b).

### Essential oil yield and plant biological yield

**Figure 5:**
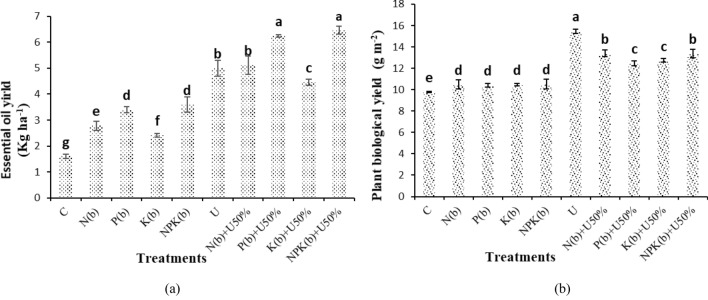
Means comparison for Essential oil yield and Plant bioiogical yield of *Nigella sativa* L. as influenced by biofertilizers and urea. Dissimilar letters show significant differences at the *p* ≤ 0.01 level. Control (C), U Chemical urea, Nb (biological N), Pb (biological P), Kb (biological K, NPKb (mixed biological NPK), Nb + U50%, Pb + U50%, Kb + U50%, and NPKb + U50%.

The highest essential oil yield was observed in (NPKb) + U50% (5.44 kg ha^−1^) and the lowest in the control (1.72 kg ha^−1^). There was no significant difference between the (NPKb) + U50% and (Pb) + U50% treatments. The (NPKb) + U50% and (Pb) + U50% treatments increased essential oil yield by 302.59 and 289.32%, respectively when compared to the control treatment (Fig. 5a).

The highest biological yield of 15.48 g m^−2^ was obtained from the treatment of U and the lowest one of 9.79 g m^−2^ from the control. The treatment of U differed from the other treatments significantly. The treatments of U and (NPKb) + U50% improved biological yield by 58% and 36.83% versus the control, respectively when compared to the control (Fig. 5b).

## Estimation of antioxidant indices

### Total phenolic compounds

The results of ANOVA revealed that all antioxidant indices were significant at the *p* ≤ 0.01 level (Table 2).

**Table 2:** The results of analysis of variance for the effect treatment on antioxidants.

Sources of variations	df	Total phenol (mg gallic acid g^−1^ DM)	Total flavonoid (mg gallic acid g^−1^ DM)	DPPH (%)	Nitric acid radical scavenging (%)	Superoxide radical scavenging (%)	Chain breaking (%)
Block	2	0.021^ ns^	0.001^ ns^	0.085^ ns^	0.348^ ns^	0.591^ ns^	0.0006^ ns^
Treatment	9	3.847**	0.087**	70.516**	153.643**	51.615**	0.229**
Error	18	0.020	0.002	0.254	0.465	0.659	0.0002
Coefficient of variations		2.655	2.718	2.044	1.654	1.543	2.445

*Ns* non-significance; *significance at the *p* ≤ 0.05 level; **significance at the *p* ≤ 0.01 level.

**Figure 6:**
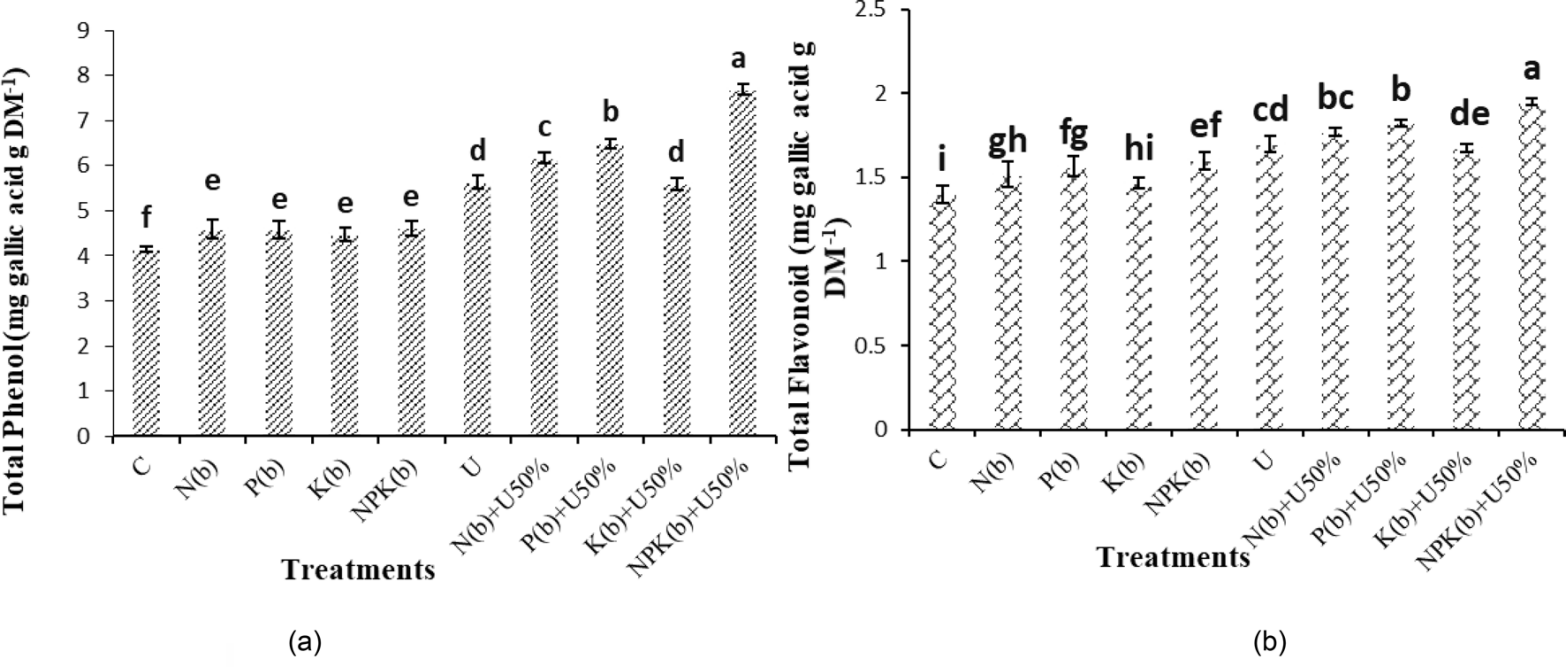
Means comparison for Total phenols and Total flavonoid of *Nigella sativa* L. as influenced by biofertilizers and urea. Dissimilar letters show significant differences at the *p* ≤ 0.01 level. Control (C), U Chemical urea, Nb (biological N), Pb (biological P), Kb (biological K, NPKb (mixed biological NPK), Nb + U50%, Pb + U50%, Kb + U50%, and NPKb + U50%.

The highest total phenol content (7.70 mg gallic acid g^−1^ dry matter (DM)) was recorded in the treatment of (NPKb) + U50%. The treatment of (NPKb) + U50% differed from the other treatments significantly. The treatments of (NPKb) + U50% and (Pb) + U50% increased total phenol content by 84.54% and 56.62% versus the control, respectively (Fig. 6a).

### Flavonoid content

The plants treated with (NPKb) + U50% showed the highest amount of flavonoids (1.95 mg gallic acid g^−1^ DM) in contrast to the control treatment with the flavonoid content of 1.40 mg gallic acid g^−1^ DM, which was the lowest. The difference of the treatment of (NPKb) + U50% with the other treatments was statistically significant. The treatment of (Pb) + U50% showed insignificant differences with the treatment of (Nb) + U50%. According to the comparison of means, the application of (NPKb) + U50%, (Pb) + U50%, and (Nb) + U50% increased total flavonoid content by 39.28%, 30.23% and 26.6%, respectively versus the control (Fig. 6b).

### Nitric oxide (NO) radical scavenging

**Figure 7:**
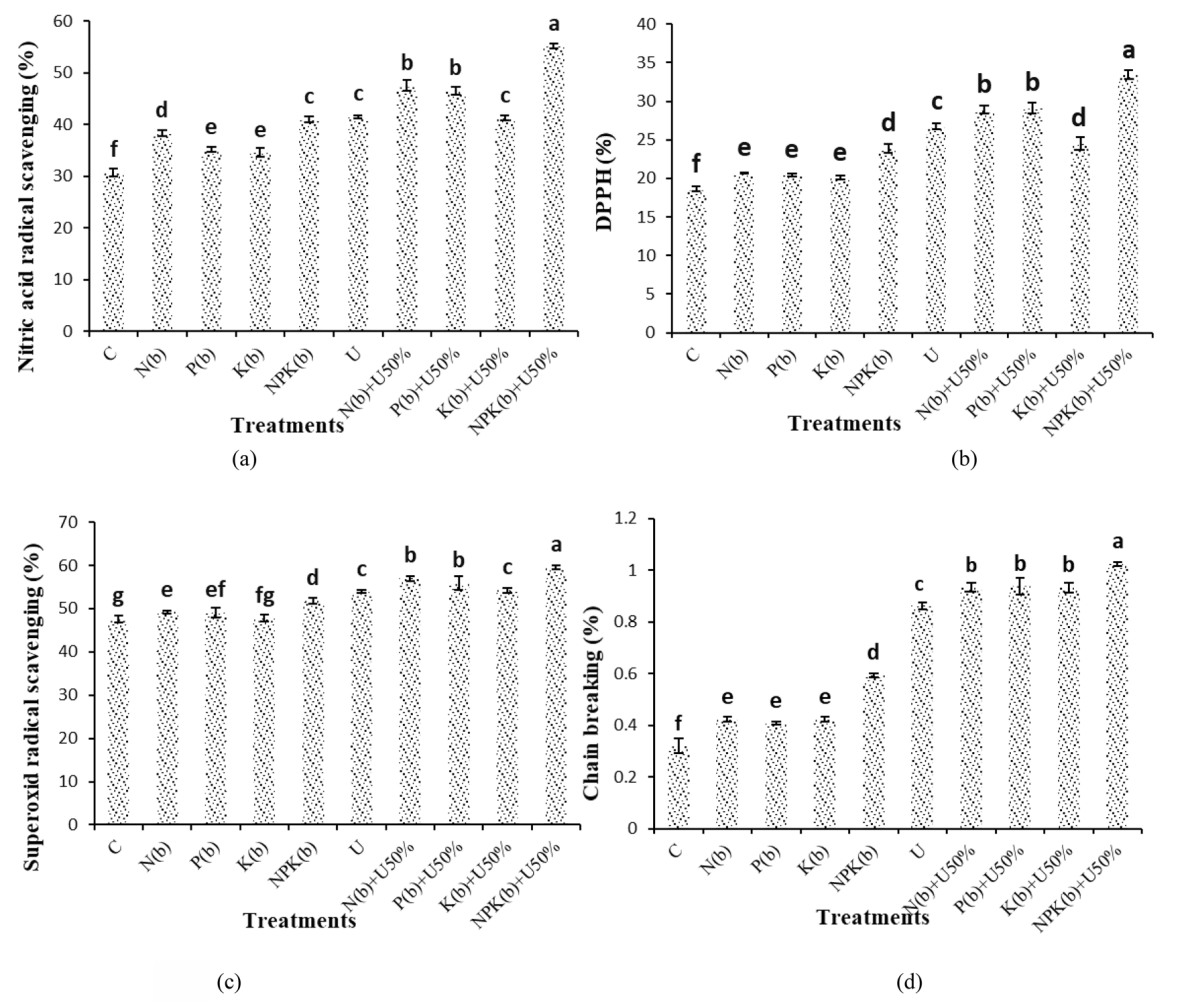
Means comparison for Nitric acid radical scavenging rate, DPPH, Superoxide radical scavenging and Chain breaking activity of *Nigella sativa* L. as influenced by biofertilizers and urea. Dissimilar letters show significant differences at the *p* ≤ 0.01 level. Control (C), U Chemical urea, Nb (biological N), Pb (biological P), Kb (biological K, NPKb (mixed biological NPK), Nb + U50%, Pb + U50%, Kb + U50%, and NPKb + U50%.

The highest and lowest levels of nitric oxide (NO) radical scavenging percentage were 55.23% and 82.30% observed in the (NPKb) + U50% and control treatments, respectively. Nitric oxide (NO) radical scavenging was increased by 79.23%, 54.35%, and 51.10% when the plants were treated with (NPKb) + U50%, (Nb) + U50%, and (Pb) + U50%, respectively (Fig. 7a).

### DPPH free radical scavenging

The plants treated with (NPKb) + U50% showed the highest DPPH free radical scavenging rate of 33.47% versus 18.63% recorded in the control plants. The difference of the treatment of (NPKb) + U50% was statistically significant from the other treatments. The plants treated with (NPKb) + U50% and (Pb) + U50% showed 79.60% and 56.35% higher DPPH free radical scavenging rate than the control, respectively (Fig. 7b).

### Superoxide radical scavenging

The treatment of (NPKb) + U50% exhibited the highest rate of superoxide radical scavenging (59.50%) as compared to the lowest (47.62%) in control. The treatment of (NPKb) + U50% differed from the other treatments significantly. Also, the plants varied insignificantly in these traits when they were treated with (Nb) + U50% or (Pb) + U50%. The comparison of the means indicated that the superoxide radical scavenging rate was increased by 24.96%, 19.63%, and 17.46% versus the control plants (Fig. 7c).

### Chain breaking activity

The (NPKb) + U50% treatment (1.02%) had the highest chain-breaking activity vis-à-vis the lowest chain-breaking activity in the control plants (0.32%). The (NPKb) + U50% treatment was significantly different from the other treatments and increased chain-breaking activity by 219.68% in comparison with the control (Fig. 7d).

### Estimation of NPK uptake in stems and leaves

NPK accumulation in leaves was significantly (*p* ≤ 0.01) influenced by the treatments (Table 3).

**Table 3:** The results of analysis of variance for the effect treatment on the accumulation of NPK in leaves.

Sources of variations	df	Leaf N percent (%)	Leaf P percent (%)	Leaf K percent (%)
Block	2	0.003^ns^	0.001^ns^	0.00001^ns^
Treatment	9	0.412**	0.010**	0.392**
Error	18	0.002	0.0006	0.002
Coefficients of variations		5.183	8.395	2.630

*ns* non-significance; *significance at the *p* < 0.05 level; **significance at the *p* < 0.01 level.

**Figure 8:**
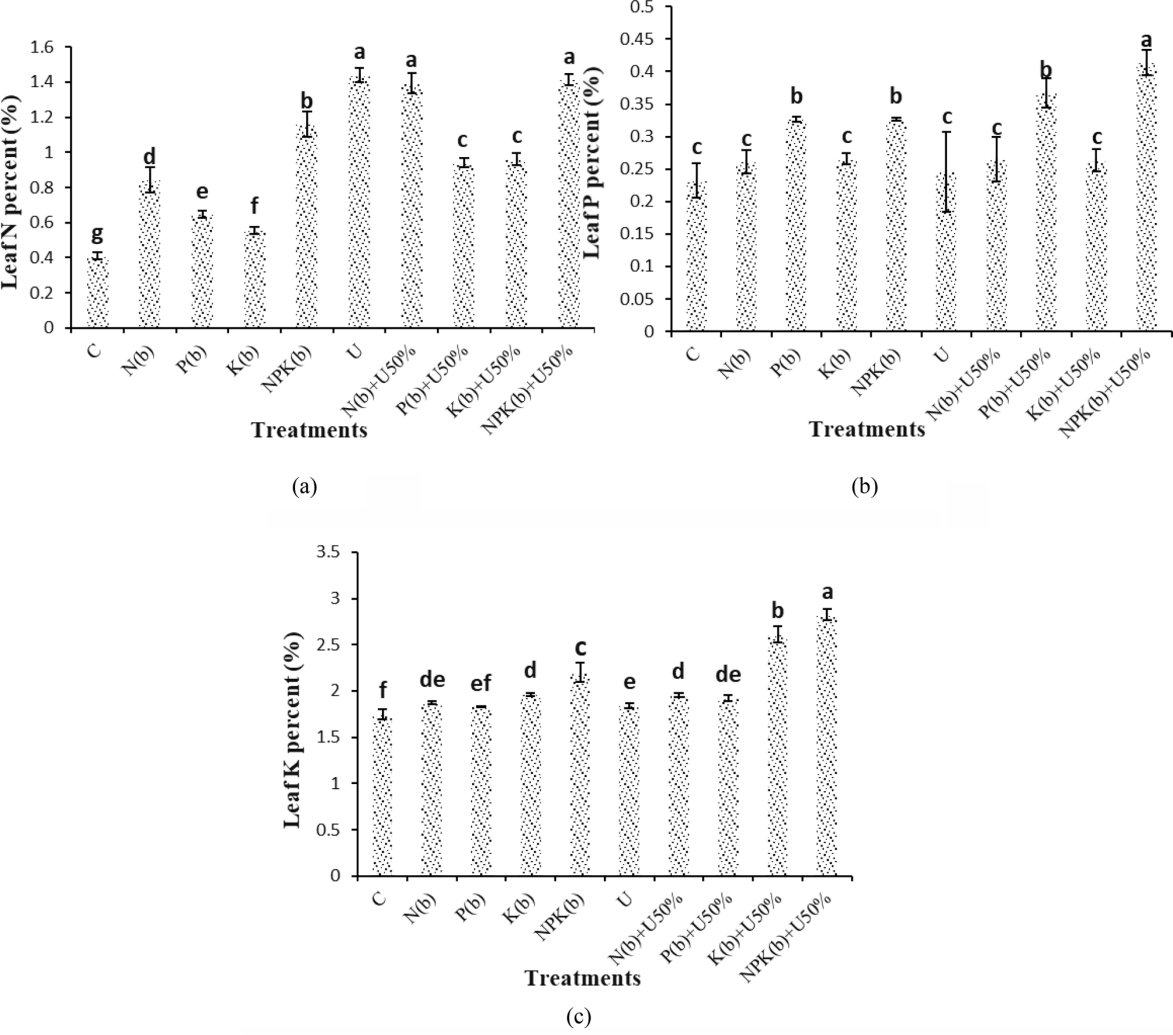
Means comparison for leaf N content, leaf P content and leaf K content of *Nigella sativa* L. as influenced by biofertilizers and urea. Dissimilar letters show significant differences at the *p* ≤ 0.01 level. Control (C), U Chemical urea, Nb (biological N), Pb (biological P), Kb (biological K, NPKb (mixed biological NPK), Nb + U50%, Pb + U50%, Kb + U50%, and NPKb + U50%.

The treatment of U resulted in the highest leaf N content (1.44%). The treatment of U did not differ from the treatments of (NPKb) + U50% and (Nb) + U50% significantly. Based on the comparison of the means, the treatments of U and (NPKb) + U50% enhanced leaf N content by 251.21% and 244.71% compared with the control, respectively (Fig. 8a).

The highest and lowest leaf P contents of 0.41% were observed in the plants treated with (NPKb) + U50%. The treatment of (NPKb) + U50% differed from all other treatments significantly. But, the difference of (Pb) + U50% with (Pb) and (NPKb) was not statistically significant. According to the comparison of the means, the treatments of (NPKb) + U50% and (Pb) + U50% resulted in 78.19% and 58.10% higher leaf P content than the control, respectively (Fig. 8b). The (NPKb) + U50% treatment resulted in the highest leaf K content (2.82%). The treatment of (NPKb) + U50% differed from the other treatments significantly. The comparison of the means revealed that the treatments of (NPKb) + U50% and (Kb) + U50% enhanced seed K content by 61.64% and 49.61% versus the control, respectively (Fig. 8c).

## Discussion

### Plant height

Many researchers have shown that biofertilizers enhance nutrient uptake by increasing root growth and development and solubilization and mineralization of soil nutrient sources such as phosphorus (P). The increase in plant height by P application can be attributed to the fact that P increases N uptake by the positive effects on root elongation^16^. Biofertilizers also stimulate the synthesis of phytohormones such as auxins, different amino acids, antibiotics, hydrogen cyanide, and siderophores. They also increase nitrogen fixation in the rhizosphere, resulting in an increase in the length of the internode and ultimately boosting the growth and height of such plants as corn and marigolds^17–22^.

Shaalan^23^ reported that when *N. sativa* L. plants were inoculated with *Azospirillum*, *Azotobacter*, and Pseudomonas, their height was increased significantly^24–26^.

### Branch diameter, capsule (follicle) number per plant, number of auxiliary branches, seed yield per plant, and thousand-seed weight

Thus far several studies have found that inoculation of seeds with biofertilizers like *Azospirillum*, *Azotobacter*, and *Pseudomonas* or the combined application of bio-chemical fertilizers increases the number of branches and capsules (follicles) per plant in *Nigella sativa* L. and plays an effective role in increasing seed yield of sesame or canola by various mechanisms such as N fixation, the synthesis of phytohormones, and different enzymes like phosphatase and biologically active compounds^27–30^. The application of biosulfurs, which contains *Thiobacillus* species, increased the seed yield of canola and sesame significantly. *Thiobacillus* as sulfur-oxidizing bacteria improved the uptake of nutrients like P^28,31,32^. Inoculation with biological fertilizers, in combined treatments, seemingly enhances the efficiency of nutrient translocation to the seed. This can be ascribed to higher photosynthetic potential and the resulting filling of the plant reproductive sinks (seeds), which in turn improves 1000-seed weight. These effects of biofertilizers or their combination with chemical fertilizers can be observed in various plants such as dill, pinto beans, and psyllium^33–36^. Also, biofertilizers have been able to provide more nutrients to the plants through bacterial secretions and pH modulation^37,38^ and have been effective in increasing production with the production of more assimilates^18^.

### Harvest index

Different harvest indexes have been reported in different plants according to the type and amount of fertilizers. It was reported that different levels of N (0, 50, 100, 150 kg ha^−1^) had a negative correlation with the harvest index of *N. sativa* L.^39^. The application of organic and biofertilizers reduced the harvest index of fennel (*Foeniculum vulgare* Mill) versus the control^40^, which is not in accordance with the results of the present study. The application of biofertilizers increased root development, water and nutrient uptake, and photosynthesis, thereby increasing the translocation of photosynthates and increasing harvest index^41^, which is in accordance with the present study. Previous research disclosed that the combined chemical and biofertilizer application showed the highest harvest index, but it did not have significant differences with Nitroxin as biofertilizer^35^.

### Essential oil percentage and essential oil yield

In a study, it was reported that phosphate-solubilizing bacteria in glandular trichomes can play a key role in enhancing the size of the trichomes and consequently increasing this element and the accumulation of essential oil^42^.

Also, the production of oils requires several raw materials, such as ATP and NADPH. The production of these substances depends on the photosynthesis of the plant, and the bacteria accelerate plant photosynthesis by providing absorbable phosphorus, which subsequently increases oil production^43^. Research has shown that high P uptake increases the amount of isopentyl diphosphate and dimethylallyl diphosphate, which is consistent with the results of this study^44^. Thus, any factor that increases nutrient uptake can affect the essential oil biosynthesis pathway and eventually lead to an increase in the essential oil content of the plant^45^. Black cumin seed contains essential oils in which Para-cymene is the main compound^46^. The results of previous studies have shown that the combined application of biofertilizers, such as nitrogen, phosphorus, and chemical (50%) fertilizers, has increased essential oil yield in basil and cumin plants^44,47^.

A study in which nitrogen-fixing and phosphate-solubilizing biofertilizers were applied to *Origanum vulgare* reported an increase in growth indices and essential oil content^48^. The application of *Azotobacter* biofertilizer to rosemary improved plant nitrogen status, thereby increasing essential oil content as nitrogen is involved in essential oil formation^49^. In a study, the highest essential oil yield per hectare and chamazulene of German chamomile were observed in the plants treated with phosphate-solubilizing bacteria and nitroxin^50^. In 2013, El-Gendy et al.^51^ reported an increase in essential oil yield in the plants treated with combined N and biofertilizers versus the control in both cultivation seasons. Volatile organic compounds are involved in a plant defense system and can be influenced by environmental microorganisms. Research has shown that the application of rhizobacteria such as Bacillus and Pseudomonas strains boosted volatile compounds content^52,53^.

### Plant biological yield

It has been reported that biofertilizers increase biological yield by enhancing useful soil rhizobia and the sustained and continuous supply of minerals including N-containing compounds to the plants^54^. It has been demonstrated that inoculation with *Azotobacter* provides appropriate conditions for plant root growth and biological functions^55^. 2018Given the positive impact of N and P on biological yields, it can be concluded that the supply of adequate P is an approach to enhancing biological yield, one of whose reasons is the vital role of P-containing compounds in energy supply in the structure of ATP because plants require much energy for N fixation^56^. In 2009, Mollafilabi et al.^39^ reported that the application of N at different rates had significant effects on increasing seed and biological yield of *N. sativa* L. Research findings revealed that integrated application of chemical, organic, and biological fertilizers ameliorated the biological yield of *Syrian cephalaria* through enhancing soil organic content, moisture, and nutrient uptake, which resulted in boosting yield components including the number of auxiliary branches^57^. Biofertilizers can have a positive effect on plant quality and quantity by facilitating the assimilation production, absorption of nutrients, and improving physiological processes^58^.

## Antioxidant indices

### Total phenolic compounds and flavonoid content

Phenols have antioxidant properties, so they scavenge and reduce reactive oxygen species (ROS), thereby preventing the oxidation of vital biomolecules of cells and avoiding oxidative stress and/or mitigating its impacts on plant cells^59^. Flavonoids possess a variety of therapeutic, biological, and pharmaceutical properties and act as antioxidants, anti-inflammation, anti-platelet, anti-thrombotic and anti-allergic^60^.

There is a large number of published studies reporting that biological fertilizers such as Rhizobium bacteria, AM (*arbuscular mycorrhiza*), and PGPR, alone or in combination with chemical fertilizers increase phenolic and flavonoid content through enhancing the interaction of soil microbes and plant roots^2,61–64^. The researchers concluded that the inoculation of tomato seeds with *Bacillus licheniformis* increased plant antioxidants and reduced the use of nitrogen fertilizer^65^. Babu et al.^66^ indicated that *Bacillus subtilis* and *B. cereus* increased peroxidase and polyphenol oxidase enzymes in tomato, which play a key role in the metabolism of phenols and flavonoids. Biofertilizers in combination or alone can increase the amount of biochemical compounds of plants, such as phenols and flavonoids, as has been seen in mung and basil plants because multi-biofertilizers usually act as growth-promoting rhizobacteria. The combination of biofertilizers with compost increases the biochemical properties of the plants compared to organic and inorganic fertilizers^67^.

### DPPH free radical scavenging assay

Colonization of mycorrhizae with roots in the presence of bacterial biofertilizers increases flavonoid content. Increasing the amount of flavonoids is correlated with DPPH radical scavenging activity. This correlation is also higher than total phenolics^68^.

Numerous studies have shown that organic and biological fertilizers alone or in combination with chemical fertilizers can increase the amount of secondary metabolites and DPPH radical scavenging activity^69–71^. Low-concentration nitrogen oxide can protect cells against oxidation, but at high levels, in combination with H_2_O_2_, it can have detrimental effects on cells, which causes toxicity. This toxicity leads to membrane damage (MDA), and DNA disorder. These toxic effects are alleviated by naturally-occurring plant extracts and antioxidants^72^. Also, broccoli extract has shown stronger antioxidant and DPPH scavenging activities when organic and biological fertilizers were applied^73^.

### Superoxide radical scavenging assay, nitric oxide (NO) radical scavenging, and chain-breaking activity

Superoxide anion radicals are produced by mitochondrial respiration. High concentrations of superoxide anions cause the formation of other ROS. These anions affect the physiological functions of cells. Plant antioxidant extracts can scavenge superoxide radicals^72^ and, as mentioned in the literature, the amount of these antioxidants can be increased by biofertilizers.

The measurement of the chain breaking activity determines the rate of radical scavenging as influenced by electron-transferring and hydrogen-donating antioxidants, which is in accordance with our results. The chain-breaking has a correlation with the amount of antioxidants that are boosted by biofertilizers. Therefore, the measurement of these parameters together can be an interesting way to estimate the antioxidant capacity of a compound^74^.

Antioxidants can mitigate the oxidative damage directly via reacting with free radicals or indirectly by inhibiting the activity or expression of free radicals. They also act as chain breakers, scavenging chain initiating radicals, quenching singlet oxygen, and chelating prooxidative metal ions^75,76^. The positive and significant correlation of phenolic compounds and flavonoids with free-radical scavenging activity is demonstrated in the turmeric rhizosphere used bio-inoculants^77^. Biofertilizers have been proven to increase the solubility of the elements and make them accessible to the roots. Also, biofertilizers increase the available iron by producing siderophores, and in general, enhance the primary and secondary metabolites of the plants, thereby increasing phenols and flavonoids, which can scavenge free radicals and also increase the inhibition of nitric oxide and superoxide radicals. Increasing the activity of the shikimic acid pathway and the activity of phenylalanine ammonia-lyase (PAL) as key enzymes in the phenol synthesis can stimulate the synthesis of phenols and flavonoids and thus inhibit radicals, in which biofertilizers play an important role^7,78,79^. In other words, antioxidants increase the inhibition of nitric oxide and superoxide radicals. These antioxidants are said chain-breaking antioxidants, which reduce the chain reaction and increase chain breakage. Hence, it can be interpreted that the combination of bio-chemical fertilizers increases the amount of antioxidants, such as phenols and flavonoids. These control the free radicals and increase primary and secondary metabolites and can play an effective role in stimulating the plant protective system.

### NPK nutrient accumulation in stems and leaves

Chemical, organic, and biofertilizers, including urea, farmyard manure, and Azetobacter, enhanced nutrient availability and root development, which resulted in better absorption of water and elements for the plant and improved plant growth^18^. The application of biofertilizers in the Thai basil plants led to the dissolution of elements in the rhizosphere of the plant and provided nutrients as evidenced by an increase in these elements in the leaves^80^. Also, the bacteria in biofertilizers can secrete a variety of hormones, such as auxin, which promotes root development and better absorption of elements^81,82^. Abo-Baker and Gehan^83^ found that a combination of chemical fertilizers, as well as phosphorus and nitrogen biofertilizers, increased the leaf P content of roselle. Also, the use of biological phosphorus fertilizers increased plant nutrition absorption and leaf nitrogen, as well as the growth parameters of tomato plants^84,85^. Concomitant use of phosphorus biofertilizer and organic fertilizer increased phosphorus in different parts of chickpea^86^. One of the main reasons for the better absorption and translocation of phosphorus can be the application of nitrogen bacteria, which was demonstrated in aonla^32^. The results of our research are consistent with the results reported from previous studies according to which the use of biofertilizers (*Azotobacter chroococcum* and *Pseudomonas fluorescens* bacteria) increases the amount of nitrogen and phosphorus in the leaves of various plants such as *Calendula officinalis* L.^87^, *Ocimum basilicum* L.^88^ and *Rosmarinus officinalis* L.^89^.

Some research has documented that the inoculation of plants with phosphate-solubilizing bacteria accelerates nutrient uptake, thereby increasing K uptake by leaves^90^. The use of azobacteria along with ammonium sulfate significantly increased the amount of potassium in corn leaves. Besides, the lowest amount of potassium in the leaf was related to the treatment without Azetobacter^55^.

The enhancement in alona leaf potassium was attributed to the combined use of organic and chemical fertilizers, which can in turn increase and improve soil properties and characteristics, resulting in well rooting and absorption of potassium. Similar results have been observed in the NPK contents of dill plants treated with combined bio-chemical fertilizers^18,91^. In other words, PGPR increases soil nitrogen fixation, nitrogen, and other plant nutrient element availability in the soil and translocation of carbohydrates from the leaves, which ultimately increases the quality and quantity of plants^92^. The results obtained from the broccoli roots inoculated with PGPR indicated an increase in nutrient content, including N, P, and K, and yield^93^.

## Conclusions

The use of biofertilizers alone or in combination with chemical fertilizer improved the quantitative and qualitative traits of black cumin. The results showed that U fertilizer increased biological yield and (Pb) + U50% enhanced essential oil percentage, while (NPKb) + U50% had the best performance in terms of plant height, branch diameter, capsule (follicle) number per plant, number of auxiliary branches, seed yield per plant, thousand-seed weight, harvest index, essential oil yield, total phenolic compounds, flavonoids content, DPPH free radical scavenging assay, nitric oxide (NO) radical, superoxide radical scavenging assay, and chain-breaking activity. In total, the use of biofertilizers and 50% urea, which reduce the use of chemical fertilizer by half, would be in line with sustainable agriculture.

## Materials and methods

### Field characteristics

The experiments were carried out in the research farm of the Department of Agriculture, Urmia University in the 2016–2017 growing season. The farm (Long. 45° 10′ E, Lat. 37° 44′ N, moisture content at field capacity: 28%, elevation 1338 m. from sea level) is located in Western Azerbaijan province, Iran.

### Soil preparation and sowing

Before the experiment, the soil was sampled from a depth of 0–30 cm for analysis (Table 4). Then, the plots were leveled and prepared in dimensions of 3 × 2.5 m^2^. Each plot was composed of 12 sowing rows spaced by 25 cm with an on-row spacing of 15 cm. The seeds were sown on March 11, 2017, and emerged with rainwater. The first irrigation was performed 2 weeks after sowing. Thinning and soil addition were performed on May 10, 2017 (thinning is the picking out the overpopulated plants in the early growing stage from a row to reach appropriate density and to ensure plants have adequate space, earthing up or ridging is the method for piling soil up around the base of a plant. This technique is used to stimulate plants growth and stabilize plants stem to avoid lodging).

**Table 4:** Physico-chemical properties of soil.

Measured trait	
Sampling depth (cm)	0–30
Salinity (ds m^−1^)	1.31
Soil texture	Loam-clay
pH	7.72
Lime (TNV) %	16.78
Clay (%)	44
Silt (%)	35
Sand (%)	21
Organic carbon (%)	0.91
Nitrogen (%)	0.03
Phosphor (mg kg^−1^)	10.33
Potassium (mg kg^−1^)	298

### Treatments

The study was carried out on the basis of a randomized block design (RBD) with 10 treatments and three replications. The treatments included control (no fertilization), U (100% chemical fertilizer as urea at 53.3 kg ha^−1^, Nb (Biofertilizer, Azotobacter vinelandii), Pb (Biofertilizer, Pantoea agglomerans and Pseudomonas putida), Kb (Biofertilizer, Bacillus spp.), NPKb (NPK, biofertilizer), Nb + 50% U, Pb + 50% U, Kb + 50% U and NPKb + 50% U. The plants were sown at a density of 40 plants per square meter or 400,000 plants per hectare. AzotoBarvar-1 contained *Azotobacter vinelandii* (strain O4), an obligate aerobic free-living gram-negative soil bacterium that fixes soil N. PhopshoBarvar-2 contained two types of phosphate solubilizing bacteria, *Pantoea agglomerans* (strain P5) and *Pseudomonas putida* (strain P13), which use the secretion of organic acids and phosphatase acids to hydrolyze insoluble P compounds. PotaBarvar-2 included two types of *Bacillus* sp. bacteria solubilizing K. The microorganisms in this fertilizer decompose insoluble K compounds in the soil around the rhizosphere and release this cation.

All biofertilizers were applied through seed priming before sowing as a recommended method. Initially, the seeds were placed in absolute darkness for 6 h in 500 ppm gibberellic acid (as priming treatment) to break dormancy and improve germination. Biofertilizers were applied as seed impregnation, and urea was mixed with soil.

### Measurements of morphological traits

Ten plants from each plot were randomly sampled to record their morphological traits on July 9, 2017, after eliminating marginal effects (marginal effects: for plant sampling, the middle rows of each plot are usually used and the side rows are not considered (eliminating marginal effects) because they are exposed to all kinds of damage, pests and diseases and can negatively affect the result of the analysis.). Plant height and branch diameter, capsule number per plant, the number of auxiliary branches, seed yield per plant, and 1000-seed weight were measured.

### Estimations of biochemical contents and biological yield

Total phenol content was stated based on mg of gallic acid in 1 g of extract employing standard quercetin curve^94^.

### Plant height, number of auxiliary branches, and number of follicles

Plant height and shoot diameter were measured with a ruler, and data on the number of auxiliary branches and follicles were collected by counting them.

To determine the seed yield of the individual plots, the moisture of the seeds collected from the follicles over an area of 1 m^2^ was adjusted to the standard level. Then, they were weighed and recorded.

### Biological yield

The plants harvested from an area of 1 m^2^ were weighed with a scale. The weight of the dry plants was regarded as the biological yield. Then, these data were used to estimate biological yield per ha.

### Essential oil content and yield

The essential oil was extracted by the method of distillation with water using a Clevenger. To this purpose, 10 g of the harvested seeds were completely crushed, poured into specific flasks, and were added with 120 mL of distilled water. The process of essential oil extraction at boiling water temperature was kept on for 4 h. Essential oil yield per unit area, which is a function of essential oil content and seed yield, was calculated by the following equation:

$${\text{Essential}} \, {\text{oil}} \, {\text{yield}} = {\text{Essential}} \, {\text{oil}} \, {\text{ content }}\left( \% \right) \times {\text{Seed}} \, {\text{ yield}}$$

### Assays on antioxidant indices

Two grams of seeds from each sample were extracted on a magnetic shaker for 3 h using 25 mL of methanol (80%) as the solvent. The resulting solution was filtered through a Whatman Grade 1 filter paper, and after it was centrifuged at 3000 rpm for 20 min, the supernatant was stored at -80 °C until the experiment day.

### Total phenols

According to this method, 1 mL of the Folin–Ciocâlteu reagent (10-fold dilution) was added to 50 µL of the extract. After 3 min, 1 mL of 10% sodium carbonate was added to the solution. The resulting solution was incubated at room temperature for 1 h after which its absorbance was read at 750 nm with an APPEL spectrophotometer^95^.

### Total flavonoid

The flavonoid content of the extract was evaluated according to the method of Zhishen et al.^96^ To determine flavonoid content, 20 µL of the plant extract was diluted with 1 mL of distilled water and was added with 0.075 mL of sodium nitrite (5%). Five minutes after the reaction, 0.15 mL of aluminum chloride (10%) was added and after 6 min, 0.5 mL of sodium hydroxide (1 mol L^−1^) was added and its final volume was adjusted to 3 mL. Then, its absorbance was read at 510 nm with a spectrophotometer.

### DPPH free radical scavenging assay

In this assay, 40 µL of the extract was mixed with 2 mL of methanolic solution (0.004%) of DPPH. The absorbance of the mixture was read at 517 nm after 30 min of incubation (at room temperature in darkness). The scavenging activity (%) of the extract was calculated by the following equation:

$${\text{DPPH}} \, {\text{ radical}} \, {\text{suppression}} \, \% =\frac{1-{A}_{sample}}{{A}_{blank}}\times 100$$

in which *A*_*blank*_ denotes the absorbance of the reaction mixture containing the extract and *A*_*sample*_ denotes the absorbance of the reaction mixture without the extract^97^.

### Nitric oxide (NO) radical

To scavenge free nitrite radicals, 40 µL of the extract was added with 0.5 mL of phosphate-buffered saline and 2 mL of sodium prusside (10 mM), and it was incubated at 25 °C for 150 min. Then, 0.5 mL of the solution was mixed with 1 mL of sulfanilic acid (10%) and was left at rest for 5 min for the reaction to be completed. Next, 1 mL of naphthyl ethylenediamine dihydrochloride (0.1%) was added and it was placed at 25 °C for 30 min until a purple color was formed in the solution. Then, its absorbance was read at 540 nm^98^.

$${\text{NO}} \, {\text{ radical}} \, {\text{suppression}} \% = \frac{\left({A}_{blank}-{A}_{sample}\times 100\right)}{{A}_{sample}}$$

in which *A*_*sample*_ represents the absorbance of the sample and *A*_*blank*_ represents the absorbance without the sample.

### Superoxide radical scavenging assay

In this assay, 9 mL of Tris-HCI buffer (pH 8.2, 50 mM L^−1^) was poured into a test tube and it was incubated in a hot bath at 25 °C for 20 min. Then, 40 µL of pyrogal solution (45 mM L^−1^ of pyrogallol in 10 mM L^−1^ of hydrochloric acid) that was already incubated at 25 °C was injected into the top part of the test tube with a 1-µL syringe and was mixed with it. The mixture was incubated at 25 °C for 3 min, immediately after which it was added with 1 drop of ascorbic acid to stop the reaction. The absorbance of the mixture at 420 nm was recorded as *A*_*0*_ after 5 min. *A*_*0*_ shows the autooxidation rate of pyrogallol. Autooxidation rate *A*_*1*_ was estimated by the same procedure. The only difference was that the Tris buffer was added only with 50 µL of the extract. At the same time, a control blank of the reaction material was considered as *A*_*2*_^96^. The radical scavenging percentage was calculated by the following equation:

$${\text{Superoxide}} \, {\text{radical}} \, {\text{suppression}} \% =\frac{\left[{A}_{0}-\left({A}_{1}-{A}_{2}\right)\right]}{A}\times 100$$

### Measurement of chain-breaking activity

The chain-breaking activity of the extracts was measured using the DPPH reagent and the protocol described in Brand-Williams et al.^99^ with slight modifications. So, 50 µL of the extract was mixed with 1.9 mL of a methanol solution of 6 × 10^–5^ M DPPH. Then, its absorbance was read at 515 nm at time 0 and 60 min after incubation at room temperature and darkness^99^. The reaction speed was estimated from the following equation:

$${Abs}^{-3}-{Abs}_{a}^{-3}=-3Kt$$

in which *K* represents the chain breaking rate, *Abs*_*a*_ represents the initial absorbance, *Abs* represents the absorbance over time *t* in minutes, and *T* represents the time in minutes.

## Estimation of NPK uptake in stems and leaves

### Nitrogen content

One gram of ground seeds was mixed with 5 g of catalyzer (copper sulfate, potassium sulfate, and copper oxide), and it was then added with 20 mL of 98% sulfuric acid. The samples were kept at 410 °C in a Kjeldahl device for 1.5 h. After they were taken out of the device, they were added with 20 mL of distilled water, and then, they were titrated with Titrasol sulfuric acid. The amount of acid applied in titration was placed in the following equation to yield nitrogen content.

$$Nitrogen \, content \left(\%\right)= \frac{Quantity \, of \, acid \, used \, in \, titration \times 0.0014}{Sample \, weight}\times 100$$

Seed P content was measured with a spectrophotometer (GEN way 630) and K content was measured with a flame photometer^100,101^.

### Phosphorus content

To measure *N. sativa* phosphorus content, 1 g of the sample was ground and meshed. After it was digested by dry burning (with HCL), it was adjusted to 100 mL by adding distilled water. Then, 5 mL of the sample was mixed with 5 mL of yellow solution (ammonium heptamolybdate + ammonium vanadate) and then, its volume was increased to 25 mL by adding distilled water. After 0.5 h, the samples were filtered through a filter paper and the resulting extract was read at 470 nm with a spectrophotometer. First, phosphorus standards and then the main samples were read. To prepare the standard, 2.19 g of KH_2_PO_4_ was first solved in a slight amount of water and was adjusted in volume in a 1-L volumetric flask (thick standard). For the series of standard solutions, 10, 8, 6, 4, 2, and 0 mL of the thick standard was taken, added with 5 mL of zinc molybdate ammonium, and adjusted to 25 mL.

### Potassium measurement

To determine *N. sativa* potassium content, 1 g of dry ground and meshed sample was placed in a furnace at 550 °C for 24 h. After digestion by dry burning method (with HCL), the samples were adjusted to 100 mL using distilled water. Using a Clinical pfp7 flame photometer, first potassium standards and then the main samples were read by the flame emission method. To prepare the standard, 9.53 g of potassium chloride was solved in water and its volume was adjusted in a 1-L volumetric flask (thick standard). Then, for a series of standard solutions, 10, 8, 6, 4, 2, and 0 mL of the thick standard was poured into 100-mL volumetric flasks containing 50 mL of water and 4.5 mL of thick sulfuric acid, and it was adjusted to the desired volume.

### Statistical analysis

Data were statistically analyzed in the SAS (ver. 9.4) software package. The means of traits were also compared by the PLSD test at the *p* < 0.05 level.

The physicochemical properties of soil in the study site (Table 4).

### Research involving plants

We wish to confirm that experimental research and field studies on plants (either cultivated or wild), including the collection of plant material, complied with relevant institutional, national, and international guidelines and legislation.
